# Hair-Cell Mechanotransduction Persists in TRP Channel Knockout Mice

**DOI:** 10.1371/journal.pone.0155577

**Published:** 2016-05-19

**Authors:** Xudong Wu, Artur A. Indzhykulian, Paul D. Niksch, Roxanna M. Webber, Miguel Garcia-Gonzalez, Terry Watnick, Jing Zhou, Melissa A. Vollrath, David P. Corey

**Affiliations:** 1 Department of Neurobiology, Harvard Medical School and Howard Hughes Medical Institute, Boston, Massachusetts, United States of America; 2 Department of Medicine, Division of Nephrology, University of Maryland, Baltimore, Maryland, United States of America; 3 Renal Division, Department of Medicine, Brigham and Women's Hospital and Harvard Medical School, Boston, Massachusetts, United States of America; 4 Department of Physiology, McGill University Montréal, Québec, Canada; University of South Florida, UNITED STATES

## Abstract

Members of the TRP superfamily of ion channels mediate mechanosensation in some organisms, and have been suggested as candidates for the mechanotransduction channel in vertebrate hair cells. Some TRP channels can be ruled out based on lack of an inner ear phenotype in knockout animals or pore properties not similar to the hair-cell channel. Such studies have excluded Trpv4, Trpa1, Trpml3, Trpm1, Trpm3, Trpc1, Trpc3, Trpc5, and Trpc6. However, others remain reasonable candidates. We used data from an RNA-seq analysis of gene expression in hair cells as well as data on TRP channel conductance to narrow the candidate group. We then characterized mice lacking functional Trpm2, Pkd2, Pkd2l1, Pkd2l2 and Pkd1l3, using scanning electron microscopy, auditory brainstem response, permeant dye accumulation, and single-cell electrophysiology. In all of these TRP-deficient mice, and in double and triple knockouts, mechanotransduction persisted. Together with published studies, these results argue against the participation of any of the 33 mouse TRP channels in hair cell transduction.

## Introduction

Sound conducted to the cochlea causes the movement of stereocilia on hair cells, the receptor cells of the inner ear. Sub-micron deflection of the bundle of stereocilia on a hair cell opens ion channels in microseconds, allowing influx of cations and the generation of a receptor potential [1,2]. Although a great deal is known about the ultrastructure and molecular mechanics of the mechanotransdution apparatus, the molecular identity of the transduction channel has been uncertain.

The physiological properties of this elusive channel provide a fingerprint for screening candidates. The transduction channel is a nonselective cation channel with high permeability to Ca^2+^ (P_Ca_/P_Na_ = 5–20) [3,4,5]. Although many divalent cations are permeant, they are also channel blockers: the channel can be blocked by Ca^2+^ (IC50 = 1 mM), Mg^2+^, La^3+^ (4 μm), and Gd^3+^ (3 μm) [6,7,8]. The single channel conductance varies considerably, ranging from about 80 to 150 pS in 2–3 mM extracellular Ca^2+^, and is roughly twice that in low Ca^2+^ [6,9,10,11,12]. Some organic cations are also permeant blockers, such as amiloride (IC50 = 50 μm [13]), the fluorescent dye FM1-43 (2 μm [14]) and the antibiotic dihydrostreptomycin (10‐70 μM [4,15,16]). The block is voltage dependent indicating that these cations block within the pore, part way along the transmembrane electric field [14,17]. Finally, transduction channels are partially permeable to large organic cations, such as choline and TEA, up to about 12 Å diameter [8]. The current view of the transduction channel shows a funnel-shaped channel with an outer vestibule and 12 Å selectivity filter [8,16,18,19]. These properties suggested that members of the transient receptor potential (TRP) family of ion channels, especially the PKD2 group, would be good candidates for the transduction channel [20,21].

The timing of gene expression provides additional clues. In mice, vestibular hair cells become mechanosensitive beginning on embryonic day 17 (E17) Cochlear hair cells show mechanosensitivity beginning between postnatal day 0 (P0) and P2, in the base and apex respectively [22,23]. We expect mRNA for the transduction channel gene to appear at or slightly before these times. Cuajungco et al. [20] analyzed expression of all 33 TRP channels in a mammalian organ of Corti library, and found 19 TRPs expressed at a single age. Asai et al. [24] went on to analyze expression of mRNAs for all TRP channels in cochlea using RT–PCR from whole inner ear tissue, at E17, E18, P0, P2, P4, P6 and P8 [24]. However, they were unable to distinguish expression in hair cells from that in supporting cells and other surrounding cells, somewhat limiting the usefulness of the analysis.

Here, we explore TRP channels as candidates for the hair cell transduction channel. We take advantage of new data on specific gene expression in hair cells at different developmental time points to narrow the candidates, and further narrow candidates by single-channel conductance and phenotypes in published TRP knockouts. With scanning electron microscopy, FM1-43 loading and single cell physiology, we investigate transduction in mouse knockouts of *Trpm2*, *Pkd2*, *Pkd2l1*, *Pkd2l2* and *Pkd1l3*, and in double and triple knockouts of these genes. We find no substantial deficit in mechanotransduction, ruling out these TRPs. Although many TRPS are expressed in hair cells and surely have important functions, we argue that none of the TRP channels are likely candidates for the transduction channel itself.

## Results

**Fig 1:**
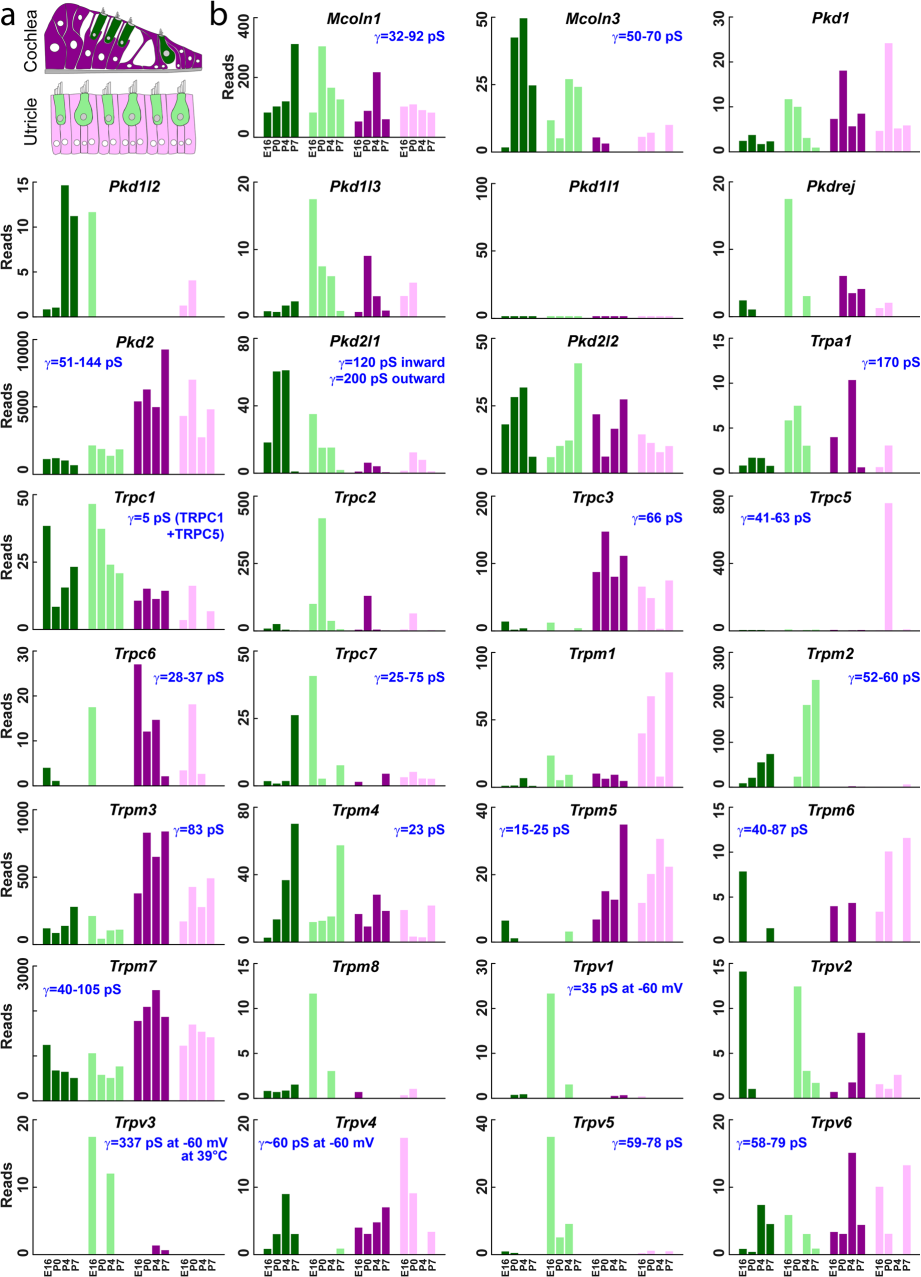
TRP channel expression in cochlear and vestibular hair cells. **(a)** Schematic of the sorted cells, redrawn from Scheffer et al., 2015 [25]. Hair cells (green) and surrounding cells (purple) were collected from the cochlea (dark colors) and utricle (light colors) at ages E16, P0, P4, and P7. **(b)** Normalized RNA-seq read counts in hair cells and surrounding cells (colors as in **a**). Data are shown for 31 of the 33 members of the TRP channel family in mouse. *Mcoln2* and *Trpc4* showed negligible counts in all samples. Data from www.shield.hms.harvard.edu [25].

To understand specific expression of TRP channels in hair cells, we queried an extensive database of gene expression in hair cells during development [25]. In that study, hair cell- and surrounding cell-specific RNA-seq libraries were prepared from FACS-sorted cochlear and vestibular tissues at E16, P0, P4 and P7. The expression of the TRP channel family for different cell types and ages is shown in Fig 1. The single-channel conductance, drawn from the literature [26,27,28,29,30,31,32,33,34,35,36,37,38,39,40,41,42,43,44,45,46,47,48,49,50,51,52,53,54,55,56,57,58,59,60,61,62,63,64,65,66,67,68] is also indicated. For reference, S1 Fig shows the TRP family in mouse arranged phylogenetically.

In narrowing candidates, we felt that an attractive transduction channel candidate should show at least two-fold enrichment in hair cells compared to surrounding cells, and it should be expressed in hair cells by the time of transduction onset. It should preferably have a large single channel conductance (>80 pS) although it is possible that a heteromeric TRP channel might have higher conductance than either subunit expressed alone. It would be more attractive if expressed in both auditory and vestibular hair cells [25] and in both inner and outer hair cells in the cochlea [69]. Knockout mice have been generated for a number of TRP channels, and we excluded all TRPs that have been reported to have normal hair cell transduction or showed no phenotype that might be expected from auditory or vestibular deficits. In mice, loss of vestibular function often leads to circling, spinning or head-bobbing behavior. Such behavior is often the first indication of an inner-ear phenotype in random or targeted mutagenesis and is usually noted in phenotypic descriptions. Auditory function is usually tested only if there is a reason to suspect dysfunction, for instance if vestibular problems are noted. Trpa1, Trpc1, Trpc3, Trpc5, Trpc6, Trpv4, Trpml3 and Trpm1 can be excluded on this basis [70,71,72,73,74]. Similarly, channels with a reported conductance <80 pS were excluded unless other strong indications were present.

### Trpm2 is not required for hair cell transduction

Based on these criteria, we first focused on *Trpm2*. Its mRNA shows 90-fold enrichment in hair cells compared to surrounding cells, and it is first expressed at E17 in vestibular system and P0 in cochlea, matching the onset of mechanosensitivity. Expression further increases during development in both tissues. Although the published conductance of 50–60 pS is somewhat below our criterion, the expression pattern warranted further investigation.

We therefore generated a conditional *Trpm2* knock-out mouse in which exon 21—encoding the fifth transmembrane and pore domains of Trpm2—is flanked by LoxP sites (*Trpm2*^fl/fl^; see Methods and S2 Fig). *Trpm2*^fl/fl^ mice were viable and could be bred as homozygotes. For hair-cell-specific Cre-mediated recombination, we crossed *Trpm2*^fl/fl^ mice with mice expressing Cre-recombinase under control of the *Gfi1* promoter [75]. PCR from genomic DNA purified from inner ears of *Trpm2*^fl/fl^:*Gfi1*-Cre^+/-^ mice confirmed the deletion.

*Trpm2*^fl/fl^:*Gfi1*-Cre^+^ homozygous knockout mice looked similar to their *Trpm2*^fl/+^:*Gfi1*-Cre^+/-^ heterozygous control littermates, suggesting no gross developmental defects. *Gfi1* is expressed in vestibular hair cells, so Cre recombination should delete *Trpm2* in these cells, however *Trpm2*-deficient mice showed no obvious vestibular deficit.

**Fig 2:**
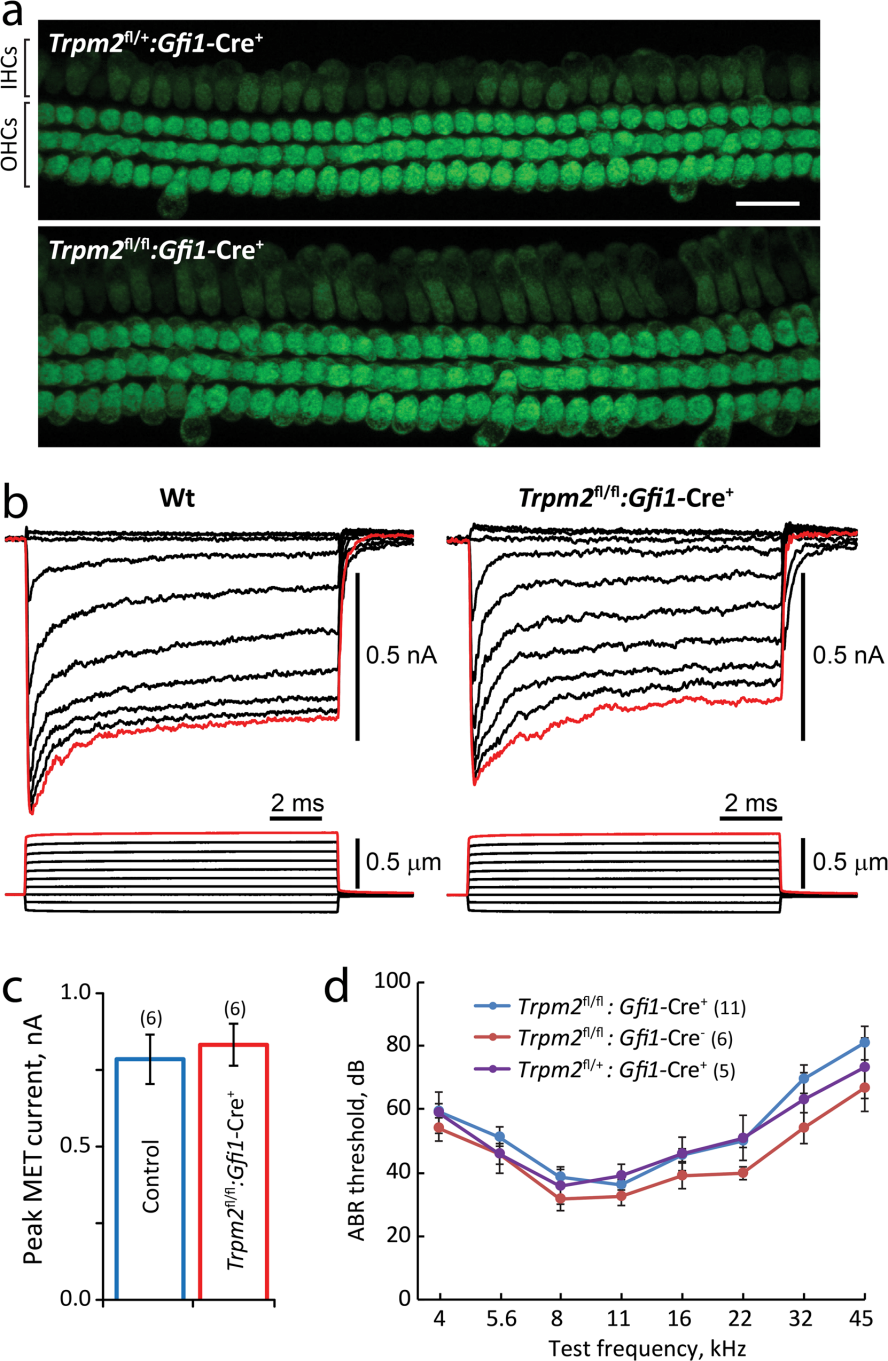
Trpm2 is not required for hair cell mechanotransduction. **(a)** FM1-43 accumulation by IHCs and OHCs from control *Trpm2*^fll+^:*Gfi1*-Cre^+^ mice *(top)* and deleted *Trpm2*^fl/fl^:*Gfi1*-Cre^+^ mice *(bottom)*. FM1-43 accumulation by hair cells was similar in control and *Trpm2*-deleted hair cells. Age: P5+2div. Scale bar = 20 μm. **(b)** A family of OHC transduction current recordings *(top traces)* in response to stereocilia bundle deflections *(bottom traces)* in WT *(left)* and *Trpm2*^fl/fl^:*Gfi1*-Cre^+^ *(right)* mice. **(c)** Average peak transduction current (red traces in **b**) for control mice (WT and *Trpm2*^fl/+^:*Gfi1*-Cre^+^) and *Trpm2*^fl/fl^:*Gfi1*-Cre^+^ mice. **d,** ABR thresholds in response to pure tone stimuli. *Trpm2*^fl/fl^:*Gfi1*-Cre^+^ mice show normal hearing. Data are mean ± sem; n as indicated.

To test mechanotransduction in *Trpm2*-deficient cochlear hair cells, we applied FM1-43 dye [76] to cultured organ of Corti explants. Both control and *Trpm2*-deleted hair cells accumulated FM1-43, showing similar levels of fluorescence intensity, suggesting that mechanotransduction persisted in the absence of *Trpm2* (Fig 2A). To further test transduction, we recorded hair cell transduction currents in response to a family of bundle deflections. Both wild-type and *Trpm2*^fl/fl^:*Gfi1*-Cre^+^ outer hair cells showed high amplitude, rapidly adapting transduction currents (Fig 2B), with peak currents in response to the largest deflections that were not significantly different (Fig 2C).

The auditory brainstem response (ABR), a sound-evoked voltage change measured near the brainstem, tests both transduction and synaptic function of the peripheral auditory system. ABR responses to pure tone stimuli were largely normal in *Trpm2*-deleted animals (Fig 2D). There was a slight elevation of hearing threshold at high frequencies in *Trpm2*-deficient mice, but overall auditory function was preserved. Based on these results we conclude that Trpm2 is not required for hair cell transduction.

### *Pkd2*, *Pkd2l1*, *Pkd2l2*, and *Pkd1l3* are not individually required for hair cell transduction

*Pkd2l1* also showed an intriguing expression pattern (Fig 1), with 6.3-fold enrichment in hair cells compared to surrounding cells in both cochlea and utricle. The reported conductance of Pkd2l1 channels, 120–200 pS [28,67], matched that expected for the transduction channel. Indeed, the PKD2 group of TRP channels has previously been suggested to include attractive candidates for the transduction channel [21].

**Fig 3:**
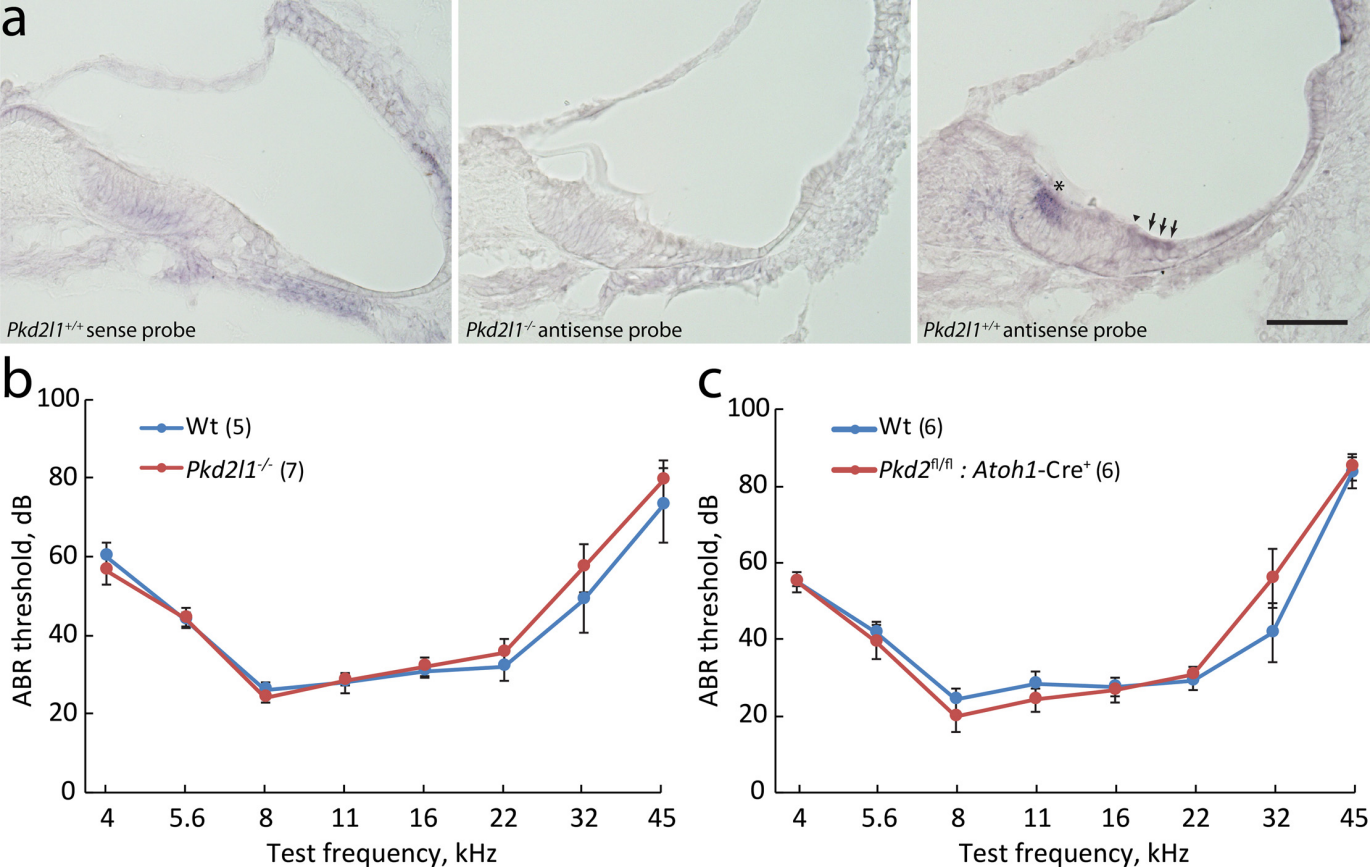
Neither *Pkd2* nor *Pkd2l1* is required for hair cell mechanotransduction. **(a)** *In situ* hybridization in cochlear sections. (*Left*) no label is evident with a control sense probe. (*Middle*) no specific label is evident in the *Pkd2l1* knockout. (*Right*) *In situ* hybridization with an antisense probe shows label of inner hair cells (arrowhead), outer hair cells (arrows) and inner sulcus cells (asterisks) in the organ of Corti. Scale bar = 50 μm; age P2. **(b)** ABR thresholds in response to pure tone stimuli. *Pkd2l1*^-/-^ mice show normal hearing at age P31-P37. **(c)** *Pkd2*^-/-^:*Atoh1*-Cre^+/-^mice show normal hearing at age 4~6 weeks. Data are mean ± sem; n as indicated.

**Fig 4:**
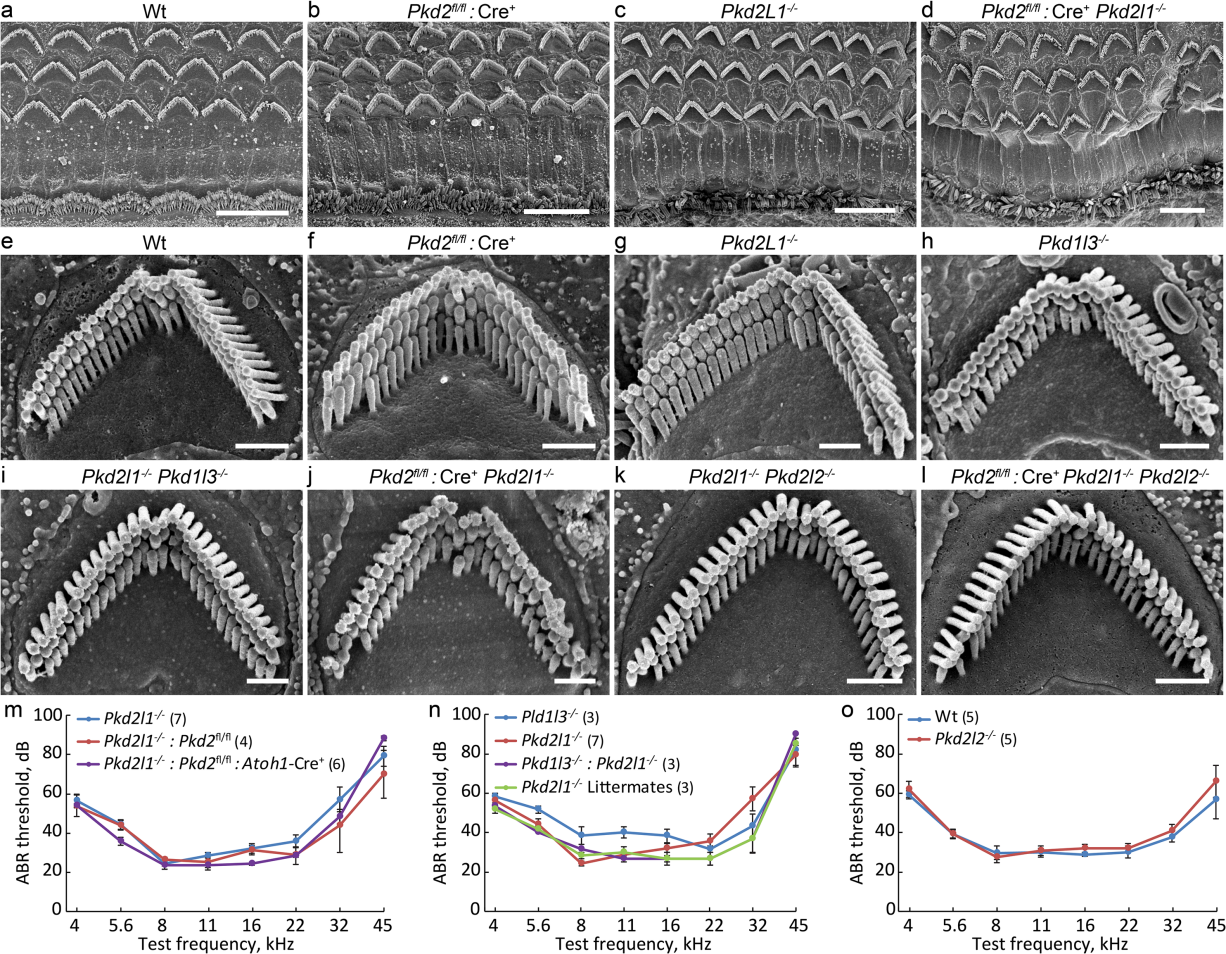
Mice lacking single or multiple PKD genes show normal stereocilia bundle morphology and hearing function. **(a-d)** SEM images of postnatal 4~6 weeks organ of Corti hair cells at low magnification in WT and PKD-deficient mice. Scale bar = 10 μm. **(e-l)** OHC bundles at high magnification. Scale bar = 1 μm. **(m)** ABR thresholds in response to pure tone stimuli in *Pkd2* and *Pkd2l1* single and double knockouts. **(n)** *Pkd2l1* and *Pkd1l3* single and double knockouts. **(o)** *Pkd2l2* knockouts. No functional deficit was observed in any combination tested. Data shown as mean ± sem.

We obtained a *Pkd2l1* knockout mouse lacking exons 3 and 4, which produced a premature stop in exon 5 [77]. Using *in situ* hybridization in cochlear sections, an antisense probe showed label in the hair-cell region (Fig 3A). No expression of *Pkd2l1* mRNA was detected in *Pkd2l1*^-/-^ mice, confirming the knockout, or with sense probe, confirming the probe specificity. However ABR measurements in *Pkd2l1*^-/-^ mice showed normal hearing thresholds (Fig 3C), indicating that mechanotransduction persists in the absence of Pkd2l1. In scanning electron microscopy (SEM) images of *Pkd2l1*^-/-^ mice at age P35, hair bundles appeared normal compared to wild-type controls (Fig 4A, 4C, 4E and 4G).

The PKD2 group of TRP channels is not very divergent (S1 Fig), and PKD2 channels interact with each other forming heteromultimeric channels, further indicating their similarity [78,79,80,81]. These both raise the possibility that expression of *Pkd2* or *Pkd2l2* might compensate for the loss of *Pkd2l1*.

We therefore generated a conditional *Pkd2* knock-out mouse line, by flanking exon 9 (aa630-671, comprising the pore and most of the sixth transmembrane domain) with loxP sites *Pkd2*^fl/fl^; see Methods and S3 Fig). Hair-cell-specific Cre recombination was achieved by crossing *Pkd2*^fl/fl^ mice with a mouse line expressing Cre recombinase under control of the *Atoh1* enhancer, restricting the Cre expression to hair cells [82]. Hair cell-specific absence of *Pkd2* expression in *Atoh1*-Cre mice was confirmed using inner ear genomic PCR (S3 Fig).

We first assessed the consequence of *Pkd2* deletion alone. *Pkd2*^fl/fl^:*Atoh1*-Cre^+^ knockout mice looked similar to their heterozygous control *Pkd2*^fl/+^:*Atoh1*-Cre^+^ littermates, suggesting no gross developmental defects. Hair bundles in *Pkd2* knockouts also appeared normal (Fig 4B–4F). ABR measurements showed normal hearing thresholds (Fig 3D), indicating that hair-cell mechanotransduction does not require *Pkd2*.

As an independent confirmation, we used a second *Pkd2*^fl/fl^ mouse line [83] missing exons 11–13, and deleted *Pkd2* in hair cells by crossing to *Atoh1*-Cre^+^. In heterozygous controls, an antibody to Pkd2 labeled the kinocilia of hair cells and the primary cilia of adjacent supporting cells (S4 Fig). In homozygous knockouts, antibody label was missing from kinocilia but not adjacent primary cilia, confirming both the antibody specificity and the cell-specific deletion. These *Pkd2* knockouts have normal hearing as well (S4 Fig).

We then obtained *Pkd2l2* knockout mice in which exons 3 and 4 were replaced with a LacZ+Neo cassette, terminated by a stop (Jackson Laboratory B6.129P2- *Pkd2l2*tm1Dgen/J, Stock #: 005829). Because Pkd2l1 was thought to function together with Pkd1l3 in acid taste transduction [84,85,86], we also obtained a *Pkd1l3* knockout line in which deletion of exons 17 through 21 produces a frame shift [87].

In double knockouts, we assessed stereocilia bundle morphology with SEM, and hearing sensitivity with ABR. Double knockout mice with hair cells missing both *Pkd2* and *Pkd2l1* showed normal bundles (Fig 4D–4J) and had normal hearing thresholds (Fig 4M). Similarly, single *Pkd1l3* knockouts and double knockouts missing both *Pkd2l1* and *Pkd1l3* showed normal bundles (Fig 4H and 4I) and normal hearing thresholds (Fig 4N). Single *Pkd2l2* knockouts are also normal (Fig 4K–4O).

### Triple *Pkd2*, *Pkd2l1* and *Pkd2l2* knockout mice show normal transduction

**Fig 5:**
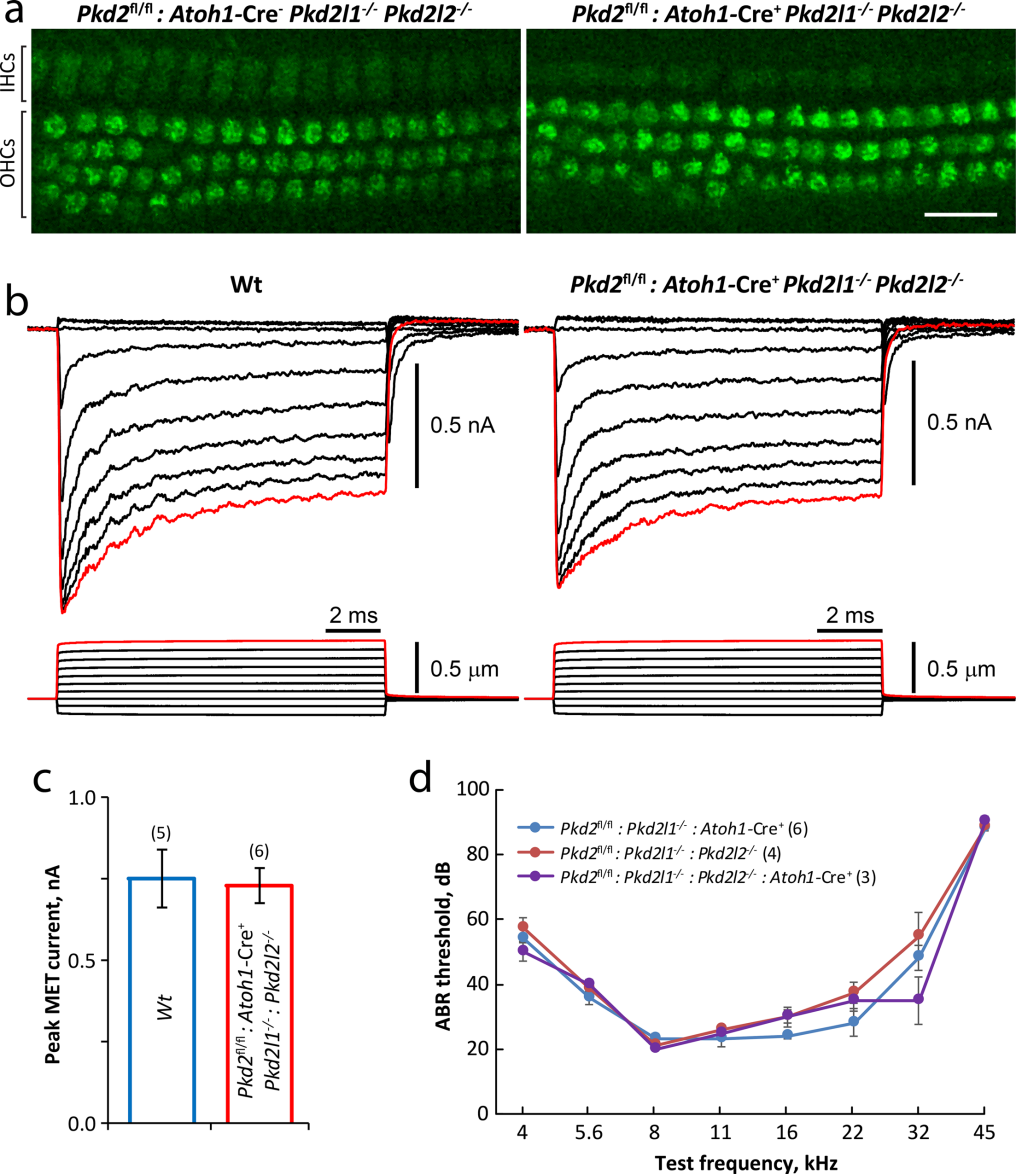
Triple PKD knockout (*Pkd2*^*fl/fl*^:*Atoh1*-Cre^+^:*Pkd2l1*^*-/*-^:*Pkd2l2*^*-/*-^) mice show normal mechanotransduction and normal hearing in ABR measurements. **(a)** FM1-43 accumulation is similar in IHCs and OHCs from P6 *Pkd2*^fl/fl^:*Atoh1*-Cre^-^:*Pkd2l1*^-/-^:*Pkd2l2*^-/-^ controls lacking Cre *(left)* and *Pkd2*^fl/fl^:*Atoh1*-Cre^+^:*Pkd2l1*^-/-^:*Pkd2l2*^-/-^ knockout mice *(right)*, suggesting normal transduction. Scale bar = 20 μm. **(b)** A family of OHC transduction currents *(top traces)* in response to stereocilia bundle deflections *(bottom traces)* in WT *(left)* and *Pkd2*^fl/fl^:*Atoh1*-Cre^+^:*Pkd2l1*^-/-^:*Pkd2l2*^-/-^ knockout *(right)* mice. **(c)** Average peak transduction currents (red traces in **b**) for WT mice (blue; n = 5) and triple knockout (red bar, n = 6). **(d)** ABR threshold measurements in response to pure tone stimuli in *Pkd2*^fl/fl^:*Atoh1*-Cre^+^:*Pkd2l1*^-/-^:*Pkd2l2*^-/-^ knockout mice indicate normal hearing. Data are mean ± sem.

*Pkd2*^fl/fl^:*Atoh1*-Cre^+^, *Pkd2l1*^-/-^ and *Pkd2l2*^-/-^ mouse lines were then crossed to each other to generate triple knockout mice. Triple knockouts were viable and could be bred as homozygotes. SEM imaging of stereocilia bundles revealed normal bundle morphology in adult mice (Fig 4L). To test transduction in cochlear hair cells missing *Pkd2*, *Pkd2l1* and *Pkd2l2*, we first used the FM1-43 dye accumulation assay in acutely dissected P6 organ of Corti epithelia. Triple knockout mice showed similar levels of fluorescence dye intensity accumulation in hair cells as compared to double knockout control mice, suggesting preserved transduction (Fig 5A). To further test transduction, we recorded transduction currents in response to a family of bundle deflections in triple knockout organ of Corti explants and in age-matched control wild-type mice of the same background. Both wild-type and *Pkd2*^fl/fl^:*Atoh1*-Cre^+^:*Pkd2l1*^-/-^:*Pkd2l2*^-/-^ OHCs showed high amplitude, rapidly adapting transduction currents (Fig 5B). The peak transduction current in response to the largest deflection was similar between the tested groups (Fig 5C). Vestibular hair cells of *Pkd2*^fl/fl^:*Atoh1*-Cre^+^:*Pkd2l1*^-/-^:*Pkd2l2*^-/-^ triple knockouts also showed transduction currents (data not shown). ABR responses to pure tone stimuli showed similar threshold levels in *Pkd2*^fl/fl^:*Atoh1*-Cre^+^:*Pkd2l1*^-/-^:*Pkd2l2*^-/-^ triple knockouts, as compared to their double knockout littermates with either *Pkd2* or *Pkd2l2* present (Fig 5D). Based on these results we conclude that the PKD2 subfamily of channels is not required for hair cell transduction.

## Discussion

The TRP channel family is broadly expressed in a different cell types and activated by a variety of stimuli, being especially prominent in reception of sensory stimuli. Like the hair-cell transduction channel, they are generally nonselective cation channels of high conductance. Some TRPs are thought to sense mechanical stimuli [88,89], although activation may be indirect [90]. This has led to the consideration of various TRPs as components of the hair cell mechanotransduction channel. Here we evaluate each of the six branches of the TRP family in mice.

### TRPMLs

The varitint-waddler mutant (Va(J)) of *Trpml3* (mucolipin 3; *Mcoln3*) has profound hearing loss [91] and impaired transduction in hair cells [64,92]. However, it was found that the Va(J) mutation leads to constitutive activation of the channel, causing a continuous inward current and consequent cell death. Hearing loss is explained by the loss of hair cells [20,61,63,64,92]. Hearing is normal in a null mutant of *Trpml3* [93].

The TRPML branch is not very divergent (S1 Fig) so there is the possibility of compensation for loss of *Trpml3* by a related gene. *Trpml2* has negligible expression in the inner ear, but *Trpml1* is expressed in both hair cells and surrounding cells (Fig 1). However the channel conductance for this branch is not consistent with the transduction channel conductance, and Trpml1 protein is mainly located in late endosomes and lysosomes [94].

### PKDs

An analysis of candidates for the hair cell transduction channel pointed out that some members of the PKD (TRPP) branch of the TRP family have properties similar to those of the transduction channel [21]. PKD2 channels are highly permeable to calcium: Pkd2 has a P_Ca_/P_Na_ of 5 whereas Pkd2l1 has a P_Ca_/P_Na_ of ~4 [95]. The portfolio of blocking ions is similar to that of hair cells: Pkd2 is blocked by Ca^2+^, La^3+^ and Gd^3+^, and Pkd2l1 is blocked by Ca^2+^, Mg^2+^, La^3+^ and Gd^3+^. Both Pkd2 and Pkd2l1 are blocked by amiloride (IC50 = 40–130 μm). The conductance of Pkd2 is 40–170 pS [40,48], and Pkd2l1 conductance is ~120 pS for inward current [67,96]. *Pkd2*, *Pkd2l1* and *Pkd2l2* are all expressed in hair cells, with the *Pkd2l1* profile being especially appropriate [25]. Moreover, some studies have suggested that the Pkd2 channel is mechanosensitive—specifically that it is located in primary cilia, where it is activated by cilia bending from environmental fluid flow [97,98,99].

Because constitutive deletion of the *Pkd2* gene is embryonic lethal, we used an *Atoh1*-Cre line to delete *Pkd2* conditionally in hair cells. Using a pure-tone ABR test, we found that hearing is normal in *Pkd2* conditional knockout mice (Fig 3D). SEM imaging showed a normal hair bundle in the cochlea. Testing a second, independent *Pkd2* conditional knockout line which truncates the C-terminal of Pkd2 channels, we found similarly normal function. Furthermore, antibody labeling showed that in hair cells Pkd2 is located in the kinocilia but not stereocilia. Finally, we recently re-analyzed mechanosensitivity in primary cilia in a variety of cell types and found that none shows rapid influx of Ca^2+^ upon cilium bending, casting doubt on the idea that Pkd2 is a mechanically gated channel [67,96,100].

We also investigated if Pkd2l1 is involved in hair cell transduction, using a *Pkd2l1* knockout mouse that we recently described [77]. RT-PCR from whole mouse cochlea showed that expression of *Pkd2l1* is correlated with the acquisition of mechanotransduction [24], and RNA-seq showed it is specifically expressed in hair cells at the start of mechanosensitivity (Fig 1). A sensitive mass spectroscopy study found that Pkd2l1 protein is enriched in hair bundles of the chicken auditory epithelium [101]. In situ hybridization in mouse showed that *Pkd2l1* is highly expressed in hair cells and also some inner sulcus cells (Fig 3A). However the *Pkd2l1* knockout mouse did not show abnormalities in hearing, using the ABR test (Fig 3C), or abnormal hair bundle morphology (Fig 4).

Pkd1l3 had been thought to form a heteromeric complex with Pkd2l1 for sour taste sensation [85,102,103], suggesting that it might share function with Pkd2l1 in hair cells. *Pkd1l3* was also found to be expressed in the auditory system [24], although expression in hair cells is limited (Fig 1). However we found that a single knockout of *Pkd1l3* and a double knockout with *Pkd2l1* showed no abnormalities in the ABR or hair bundle morphology (Fig 4).

Pkd2l2 is also expressed in hair cells, although not preferentially (Fig 1). We found that a single *Pkd2l2* knockout has a normal hearing sensitivity and hair bundle morphology. Because of the possibility of compensation among this closely-related group, we created double and triple knockouts of *Pkd2*, *Pkd2l1* and *Pdk2l2*. Even triple knockouts showed no deficit in hearing sensitivity, bundle morphology, or mechanotransduction assessed with FM1-43 accumulation and single-cell recording. We can thus rule out the PKD2 branch of the TRP family.

In addition to *Pkd1l3*, some of the *Pkd1* group of TRPs are sparsely expressed in hair cells. However none of the *Pkd1* group have robust and hair-cell-specific expression profiles (Fig 1). *Pkd1* mutants have normal transduction current and only moderate loss of hearing sensitivity [104]. Moreover, PKD1s are thought to form ion channels as heteromultimers with PKD2 channels [105], and the PKD2s have been ruled out.

The entire PKD group of TRP channels is thus unlikely to participate in hair-cell transduction.

### TRPA

Trpa1 was also considered as a strong transduction channel candidate because knockdown of expression in both mice (with virally delivered siRNA) and zebrafish (morpholino injection) reduced the transduction current, and because it has a large single-channel conductance of 250 pS in low Ca^2+^ [106], (data not shown). However, mice lacking *Trpa1* have normal transduction current and hearing [70]. *Trpa1* is the only member of the *Trpa* branch in mice, so compensation by a related gene is unlikely.

### TRPCs

Although *Trpc1* is expressed in mouse hair cells (Fig 1), the other members of this branch do not have an appropriate expression pattern and *Trpc4* is not expressed at all. In addition, a quadruple knockout of *Trpc1*, *Trpc3*, *Trpc5* and *Trpc6* shows normal hearing sensitivity and hair cell morphology [71,72]. We previously showed that the remaining TRPC gene, *Trpc2*, is highly expressed in mouse vomeronasal neurons and the protein is located in the sensory cilia where it is thought to participate in pheromone transduction [107]. *Trpc2* knockout mice do lack pheromone sensitivity but have no vestibular deficit [108]. Also, *TRPC2* is a nonfunctional pseudogene in a species (human) that has normal hearing [109]. Finally, the single channel conductance of the TRPC channels is lower than that of the hair cell transduction channel.

### TRPMs

In situ hybridization and RNA-seq showed *Trpm*2 to be selectively expressed by inner ear hair cells, suggesting that it plays an important role in these cells [25]. We found, however, that *Trpm*2 knockout mice exhibited normal transduction current and FM-143 accumulation. *Trpm*1 knockouts also have normal hearing [73]. It might be that other TRPMs compensate in these knockouts, but—with the exception of *Trpm*7—none of the others has an appropriate expression pattern or single-channel conductance. *Trpm*7 is expressed but not enriched in hair cells, its single channel conductance is high but not as high as the transduction channel, and zebrafish studies suggest it is not required for hair cell transduction in zebrafish [110]. Thus none of the TRPMs seems likely to be a transduction channel.

### TRPVs

Trpv4 was first suggested as a possible component of a heteromeric transduction channel because it was mechanosensitive—responding to changes in osmolarity—and was expressed in auditory hair cells [111]. Newer methods have shown the *Trpv4* gene to be expressed only at very low levels in cochlear hair cells and not at all in utricular hair cells [25]. Although *Trpv4* knockout mice show mild, late onset hearing loss, there is no deficit in hearing at earlier ages, excluding it as a transduction channel candidate [72].

Of other TRPVs, only Trpv3 has a large conductance; however its expression pattern is not consistent with the hair cell transduction channel (Fig 1). None of the other TRPV channels shows an appropriate expression pattern in hair cells.

### Conclusion

Although the TRP family of ion channels initially seemed like a rich source of candidates for the hair-cell transduction channel, most of them can be ruled out by inappropriate expression pattern, inappropriate conductance, or lack of an auditory or vestibular phenotype in knockout mice. Here, we have tested the remaining reasonable candidates—including some that were quite attractive based on expression and conductance—but found no deficit in transduction in hair cells of these animals. Although many TRPs are expressed in hair cells and many are sure to carry out important functions in these cells, it now seems safe to exclude the entire TRP family in the search for a transduction channel.

## Materials and Methods

### TRP channel expression data

Expression data for all 33 mouse TRP channels were drawn from Scheffer et al., 2015, as posted on the SHIELD database (shield.hms.harvard.edu). Read counts were analyzed based on the 20207 RefSeq genes in the DNAnexus set. Pkd1l1 is not in that set, so reads for Pkd1l1 were identified by specifically querying the raw data set.

### Mouse lines

This study was carried out in strict accordance with the recommendations in the Guide for the Care and Use of Laboratory Animals of the National Institutes of Health. The protocol was approved by the Institutional Animal Care and Use Committee at Harvard Medical School (Protocol Number: 03524). ABR measurements were performed under anesthesia (ketamine (100 mg/kg)/xylazine (10 mg/kg) cocktail). Newborn mice (P0-P4) were anesthetized by cooling, then euthanized by decapitation. Adult mice were euthanized by isoflurane overdose, followed by cervical dislocation. All efforts were made to minimize suffering. All primers used in current study are summarized in **Table 1**.

**Table 1:** Primers used for genotyping and validation of gene deletion.

Mouse lines	Primer Name	PCR primer concentration	Sequence	Floxed allele	WT allele	KO allele	Purpose
***Trpm2*cKO (David Corey)**	LOX	1.2 μM	TGAGGCGGAAGGAATTAGCAC	320	259		Genotyping
	SDL	1.2 μM	CCCACCTGACAGTCACAAGTGTG	320	259		Genotyping
	TM2cKO15101f	1.2 μM	GACTTCATCATGTTCTGTCT	4184 (N/A*)	2415 (N/A*)	616	Verification cKO in the inner ear
***Pkd2*cKO (Jing Zhou)**	mPKD2in9F3	0.6 μM	TTGTGCATTTGGTGATGTGTTA	520	468		Genotyping
	mPKD2in9R3	0.6 μM	CCACATTTACATGGCATCTGAG	520	468		Genotyping
	PKD2-5940f1	1.2 μM	AAGCTGTGTTATCATTCTAGAAAGC	>2457	2457	344	Verification cKO in the inner ear
***Pkd2*cKO (Terry Watnick)**	MG/3 flox c-f	1.2 μM	GGGGTTTCCTATGAAGAGTTCCAAG	485	396		Genotyping
	MG/3 flox d-r	1.2 μM	CTGACAGGCACCTACAGAACAGTG	485	396		Genotyping
***Pkd2l1***	Neo82	0.6 μM	CTGCCTTGGGAAAAGCGCCT		~520	~480	Genotyping
	MPclF904	0.6 μM	AAGATCAGCTCCCCTTTGGACCT		~520	~480	Genotyping
	R520	0.6 μM	TTCCACCCCAGGATTCTCTG		~520	~480	Genotyping
***Gfi1*-Cre**	Gfi-1F	1.2 μM	GGG ATA ACG GAC CAG TTG		609	672	Genotyping
	Gfi1Cre-R	1.2 μM	GCC CAA ATG TTG CTG GAT AGT		609	672	Genotyping
	Gfi-1R	0.6 μM	CCG AGG GGC GTT AGG ATA		609	672	Genotyping
***Atoh1*-Cre**	Atoh1Cre-F	1.2 μM	ATCGGCCTCCTCCTCGTAGACAGC			550	Genotyping
	Atoh1Cre-R	1.2 μM	GGATCCGCCGCATAACCAGTGA			550	Genotyping
***Pkd2l2***	IMR4245	0.3 μM	CATCATCAGGTAGAGAAGTGTCCAC		~200	~450	Genotyping
	IMR4246	1.2 μM	CGTGCGTGCAAACACCCACACACAG		~200	~450	Genotyping
	IMR5100	1.2 μM	TTCAACAGACCTTGCATTCCTTTGG		~200	~450	Genotyping
***Pkd1l3***	1111nF-5’	0.3 μM	AGGAGAGGATTGACTTCTATGAG		~250	650	Genotyping
	1112nR-5’	0.3 μM	CAGGAGAGCCTCTGGACTCGTGT		~250	650	Genotyping
	1328F-5’	1.2 μM	GATGGAAGCCGGTCTTGTCGAT		~250	650	Genotyping
	1538R-5’	1.2 μM	TCGAGCCCCAGCTGGTTCTTTCC		~250	650	Genotyping

* Not amplified

#### *Trpm2* conditional knockout mouse line

We worked with Ingenious Targeting Laboratory (Ronkonkoma, New York, USA) to make the *Trpm2* conditional knockout. A 10.2-kb *Trpm2* genomic fragment was subcloned from a C57BL/6 BAC clone to construct the targeting vector using homologous recombination. A FRT-LoxP-Neo-FRT-LoxP cassette was inserted upstream of exon 21 and the third single LoxP site was inserted downstream of exon 21 (S2 Fig). The region flanked by the second and the third LoxP sites is about 2.0 kb. Exon 21 encodes amino acids 929–985 and corresponds to the fifth transmembrane domain and pore of *Trpm2*; its deletion also causes an early stop. A homology short arm extends 1.8 kb to the 3’ of the FRT-LoxP-Neo-FRT-LoxP cassette whereas a homology long arm extends 6.4 kb from the 5’ side of the third LoxP site. The linearized targeting vector was then electroporated into iTL BA1(C57BL/6X129/SvEv) hybrid embryonic stem cells.

#### *Pkd2l1* constitutive knockout

We have previously described the *Pkd2l1* knockout [77]. It lacks exons 3 and 4, leading to a premature stop codon in exon 5.

#### *Pkd2* conditional knockout

A 7.3kb genomic fragment of the *Pkd2* gene was used to construct the targeting vector. Three LoxP sites were inserted as shown in S3 Fig. The first LoxP site was inserted upstream of exon 9 and a LoxP-Neo-LoxP cassette was inserted downstream of exon 9. The first and second LoxP sites thus flank a 2.2kb genomic region that includes exon 9. Exon 9 encodes amino acids 630–671, comprising the pore and most of the sixth transmembrane domain of Pkd2. The homology short arm extended 0.9 kb to the first loxP site and a homology long arm extended 4.2 kb downstream of the LoxP-Neo-LoxP cassette.

#### *Pkd2l2* constitutive knockout

Mice were obtained from The Jackson Laboratory (B6.129P2-*Pkd2l2*^tm1Dgen/^J, #005829). In this mutant mouse line, a lacZ-Neo cassette was inserted into the *Pkd2l2* gene to replace a 1.8-kb genomic fragment including exons 3 and 4. As a result, the endogenous *Pkd2l2* gene promoter drives expression of the beta-galactosidase gene but not the endogenous *Pkd2l2* gene (S5 Fig).

#### *Pkd1l3* constitutive knockout

Mice were obtained from The Jackson Laboratory (B6;129S4-*Pkd1l3*tm1Sul/J, Stock #: 008419). They lack exons 17 through 21, which encode transmembrane domains 2–5 [87].

#### Cre lines

The *Gfi1*-*Cre* mouse line was generously provided by Dr. Lin Gan (University of Rochester)[75]. The *Atoh1*-*Cre* mouse was obtained from The Jackson Laboratory (B6.Cg-Tg(*Atoh1-cre*)1Bfri/J, #011104) [82].

### Immunofluorescence

Mouse cochleas and/or utricles were dissected from P2~P8 mutant mice and their wild-type littermates. Samples were fixed with 4% formaldehyde for 2 hr, rinsed with PBS, incubated in 0.1 M citrate buffer (pH 7.0) at 60°C for 30 min for antigen retrieval. Whole mount samples were permeabilized with 0.5% Triton X-100 for 30 min, and blocked with 10% goat serum supplemented with 0.5% Triton X-100 for 30 min. Samples were then incubated with primary antibody overnight at 4°C, rinsed with PBS, and further incubated in secondary antibodies together with phalloidin for 6 hr. Samples were then mounted with Prolong Gold antifade kit (Invitrogen), cured in the dark at room temperature overnight and imaged with an upright Olympus FluoView FV1000 confocal laser scanning microscope (60X 1.42NA objective).

### ABR measurements

The ABR assay was performed using a Tucker Davis Technologies (TDT, Gainsville, FL) workstation (System III). Mice age P28 to P60 were anesthetized by intraperitoneal injection of a ketamine (100 mg/kg)/xylazine (10 mg/kg) cocktail. Anesthetized mice were then placed on a heating pad and electrodes were placed subcutaneously in the vertex, underneath the left ear, and on the back near the tail. Tone stimuli of 4, 5.6, 8, 11.2, 16, 22, 32 and 45.3 kHz were calibrated with a precision microphone system (PS9200Kit, ACO Pacific, USA) using the TDT SigCal software package. The recorded signals were band-pass filtered (300 Hz to 3 kHz) and amplified 100,000 times. The number of acquisition trials was set to 500 averages. Maximum stimulus intensity was set to 95 dB peak SPL with attenuation decreasing from 85 dB to 0 dB SPL at 5 dB SPL intervals. All ABR thresholds were read by second investigator who was blind to the mouse genotype.

### Field emission scanning electron microscopy

Cochleas from either P2 or adult (P28-P40) mice were dissected out and immediately immersed in 0.5% glutaraldehyde / 0.1 M sodium cacodylate buffer / 3 mM CaCl_2_ (pH 7.3) for 2 hr. Adult cochleas were further decalcified in 120 mM EDTA (pH 7.2) for 24 hr. Cochlea coils were dissected out in distilled water. Samples were processed through an OTOTO procedure [112] with modifications. Briefly, samples were washed in the cacodylate dilution buffer three times for 5 min each, fixed in 1% OsO_4_ in cacodylate buffer for 1 hr, and washed in H_2_O for 5 min three times. The samples then went through 1% freshly prepared tannic acid for 1 hr, then 1% OsO4 for 1 hr, 1% tannic acid for 1 hr, and 1% OsO_4_ for 1 hr, with an H_2_O wash between steps. Next, samples were washed and processed through the crescent isopropanol series steps (30% isopropanol at room temperature, 50% isopropanol on ice, 70% isopropanol at -20°C, 90% isopropanol slurry on dry ice, 95% isopropanol slurry on dry ice, 100% isopropanol slurry on dry ice), with each step exposition set to 15 min. Sample were then critical point dried, sputter coated with platinum and imaged on a Hitachi S-4800 field emission scanning electron microscope.

### Whole-cell patch clamp recording

Organ of Corti epithelia were dissected at P3-P5 in L-15 medium (Life Technologies), placed in glass bottom Petri dishes (WPI Inc.) and cultured for 2–3 days in DMEM/F12 medium (Life Technologies) supplemented with 5% FBS and 10 mg/l ampicillin at 37°C (10% CO_2_) as previously described [113]. Experiments were performed at room temperature in L-15 containing the following inorganic salts (in mM): NaCl (137), KCl (5.4), CaCl_2_ (1.26), MgCl_2_ (1.0), Na_2_HPO_4_ (1.0), KH_2_PO_4_ (0.44), MgSO_4_ (0.81). Hair cells were observed with an inverted microscope (TE 2000, Nikon) using a 100X 1.3 NA oil-immersion objective lens and DIC optics. Pipettes for whole-cell patch-clamp recordings were filled with intracellular solution containing (in mM): CsCl (140), MgCl_2_ (2.5), Na_2_ATP (2.5), EGTA (1.0), HEPES (5.0). The pipette resistance was typically 4–6 MΩ when measured in the bath. Patch clamp recordings were performed with an AxoPatch 200B amplifier (Molecular Devices) controlled by pClamp 9 software package. Hair cells were held at –60 mV between the short periods of transduction recordings, when the holding potential was temporarily hyperpolarized to –90 mV. All recorded hair cells were located 35–45% away from the apex of the organ of Corti explant.

Hair bundles were deflected using a stiff glass probe, fire-polished to fit the shape of the stereocilia bundle (~5–7 μm). The probe was mounted on a piezo actuator (PA 8/14 SG, Piezosystem Jena), equipped with a strain gauge sensor to provide a direct reading of the probe’s axial displacement. The piezo was driven by a custom-made amplifier, providing a rapid step deflection within ~40 μs (10–90% risetime). The angle between the axis of the probe movement and the bottom surface of a dish was kept constant at ~30 degrees.

### FM1-43 loading

Organ of Corti epithelia were acutely dissected from P4-P5 mice in L-15 cell culture medium, and either mounted on coverslips using tungsten minutien pins (WPI Inc.) for the experiment, or cultured for an additional 2 days as described above. Following tectorial membrane removal and medium aspiration, FM1-43 solution (2 μM in L-15) was applied to the tissue for 30–60 s, then quickly aspirated; the tissue was rinsed once with L-15 and the excessive dye quenched by a 0.2 mM solution of 4-sulphonato calix[8]arene, sodium salt (SCAS, Biotium) in L-15. The organ of Corti was then observed on an upright Olympus FV1000 confocal microscope, equipped with 60X 1.1 NA water-dipping objective lens.

## Supporting Information

S1 Fig Mouse TRP channel phylogeny. There are 33 genes in six major groups (TRPM, TRPC, TRPV, PKD/TRPP, TRPML and TRPA). Length of line indicates divergence.
(TIF)

S2 Fig Strategy and validation of *Trpm2* knockout. **(a)** *Trpm2* conditional knockout strategy. We inserted two LoxP sites to flank a ~1.9 kb region that includes exon 21. A LoxP-FRT-Neo-LoxP-FRT cassette was inserted upstream of exon 21 and the third LoxP site was inserted downstream of exon 21. Exon 21 encodes aa929-985 including the essential fifth transmembrane domain and pore. The deletion also led to a downstream frameshift. Red arrowheads refer to LoxP sites, yellow brackets refer to FRT sites, and green arrows refer to the neomycin resistance gene. Primers used for genotyping and validation are indicated as gray arrows. **(b)** PCR from genomic DNA purified from inner ears of *Trpm2*^fl/fl^: *Gfi1*-Cre^+^ mice, *Trpm2*^fl/fl^:*Gfi1*-Cre^-^ mice, and an age-matched wildtype mouse. *(Left)* PCR results using primer pair TM2cKO15101f and SDL. Lane 1 (*Trpm2*^fl/fl^:*Gfi1*-Cre^+^) shows a fused short band (616bp) only from the deleted allele; lane 2 lacking Cre (*Trpm2*^fl/fl^:*Gfi1*-Cre^-^) and Lane 3 (wildtype) do not show it. The fused band was confirmed by Sanger sequencing. (*Middle*) PCR bands at 320 bp (floxed allele) in lane 1 and lane 2, and 259 bp (Wt allele)in lane 3, using primers LOX and SDL, (*Right*) Genotyping for the *Gfi1*-Cre allele. A 1-kb DNA extension ladder was used (5 μl; Invitrogen #10511–012).
(TIF)

S3 Fig Strategy and validation of *Pkd2* knockout. **(a)** Exon 9 of the *Pkd2* gene was deleted by flanking the ~2.2 kb targeting region with two LoxP sites. A LoxP-Neo-LoxP cassette was inserted downstream of exon 9 and the third LoxP site was inserted upstream. Exon 9 encodes aa630-671 including the pore domain and most of the sixth transmembrane domain of the Pkd2 channel. Primers used for genotyping and validation are indicated as a gray arrows. **(b)** PCR from genomic DNA purified from inner ears of *Pkd2*^fl/fl^:*Atoh1-Cre*^+^ mice, *Pkd2* ^fl/fl^:*Atoh1-Cre*^-/-^ mice, and an age-matched wildtype mouse. *(Left)* PCR results using primer pair PKD2-5940f1 and mPKD2in9R3. Lane 1 (*Pkd2*^fl/fl^:*Atoh1-Cre*^+^) shows a fused short band (344 bp) from the deleted allele and a longer band (>2437 bp) from the genomic DNA cells without Cre activity. The short fused band was confirmed by Sanger sequencing. Lane 2 (*Pkd2* ^fl/fl^:*Atoh1-Cre*^-^) shows the same long band as in lane 1 but no short band. Lane 3 shows a band of 2457 bp produced from a wildtype mouse inner ear. (*Middle*) PCR produced bands at 520bp in lane 1 and lane 2, and 468 bp in lane 3 using mPKD2in9F3 and mPKD2inR3. (*Right*) Genotyping of the *Atoh1-Cre* allele. A 1-kb DNA extension ladder was used (5 μl; Invitrogen #10511–012).
(TIF)

S4 Fig Pkd2 localization and *Pkd2* knockout ABR data, in the second *Pkd2* knockout mouse [83]. **(a,a’)** Antibody labeling (Santa Cruz Biotechnology, #sc-10376) for Pkd2 (red) and phalloidin staining for actin (green) in the heterozygote (*Pkd2*^fl/+^: *Atoh1*-Cre^+^) positive-control cochlea. Pkd2 label is evident in hair-cell kinocilia (arrowheads) and some supporting cell primary cilia (arrows). **(b,b’)** *Pkd2*^fl/fl^: *Atoh1*-Cre^+^ knockout cochlea. Pkd2 label is absent from hair cell kinocilia but not from supporting cell cilia. **(c-c’)** In vestibular hair cells of heterozygote mice, Pkd2 label is also in kinocilia. **(d)** ABR thresholds in response to pure tone stimuli. *Pkd2*^fl/fl^: *Atoh1-Cre*^+^ knockout mice show normal hearing. Data are mean ± SEM.
(TIF)

S5 Fig *Pkd2l2* knockout. The generation of the *Pkd2l2* knockout mouse, based on information from The Jackson Laboratory (*Pkd2l2*^*tm1Dgen*^/J; stock #005829; https://www.jax.org/strain/005829). A bacterial lacZ gene fused with a neomycin resistance gene replaced ~ 1.8 kb genomic sequence extending from the 3’ part of exon 3 to the 5’ part of exon 4. Thus the endogenous promoter drove the expression of beta-galactosidase.
(TIF)
