# Supplementary figures and images for: Hair-Cell Mechanotransduction Persists in TRP Channel Knockout Mice
Source: PLoS One. 2016 May 19;11(5):e0155577. doi: 10.1371/journal.pone.0155577 (PMC4873267; doi:10.1371/journal.pone.0155577)

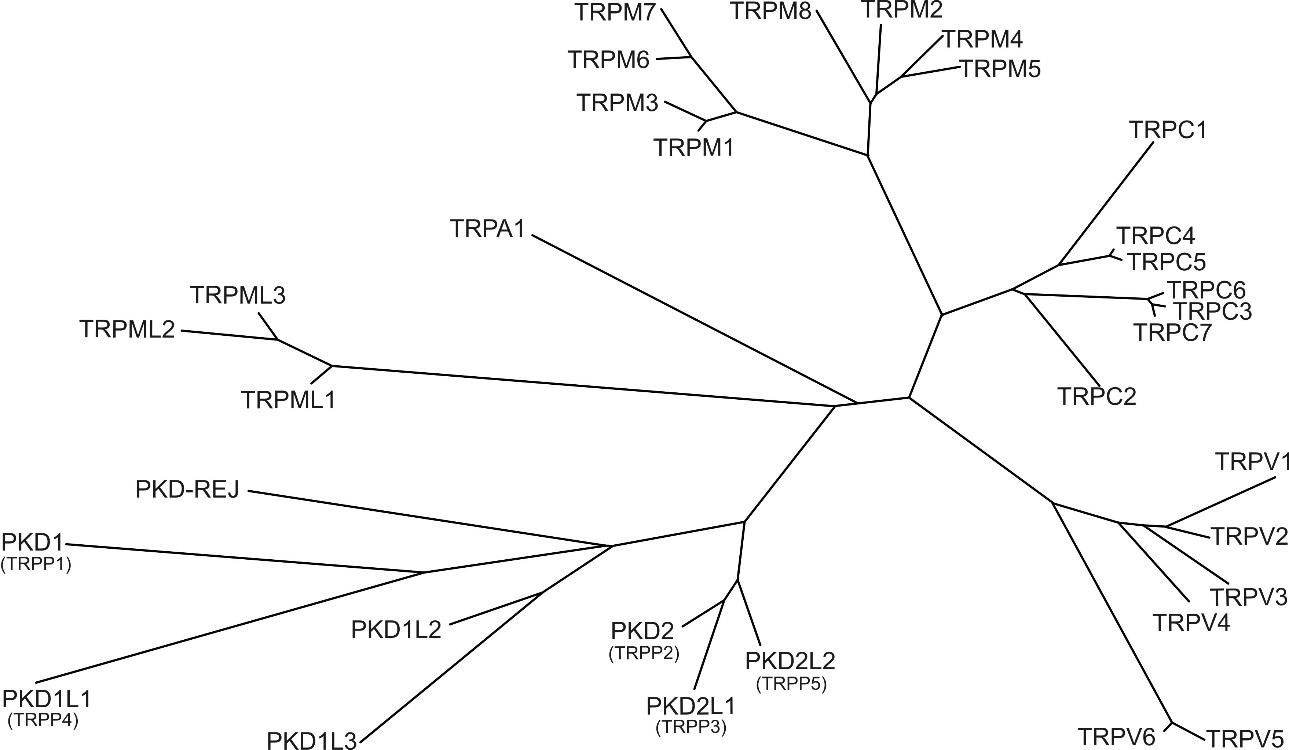

Supplement: S1 Fig — There are 33 genes in six major groups (TRPM, TRPC, TRPV, PKD/TRPP, TRPML and TRPA). Length of line indicates divergence. (TIF) [file pone.0155577.s001.tif]

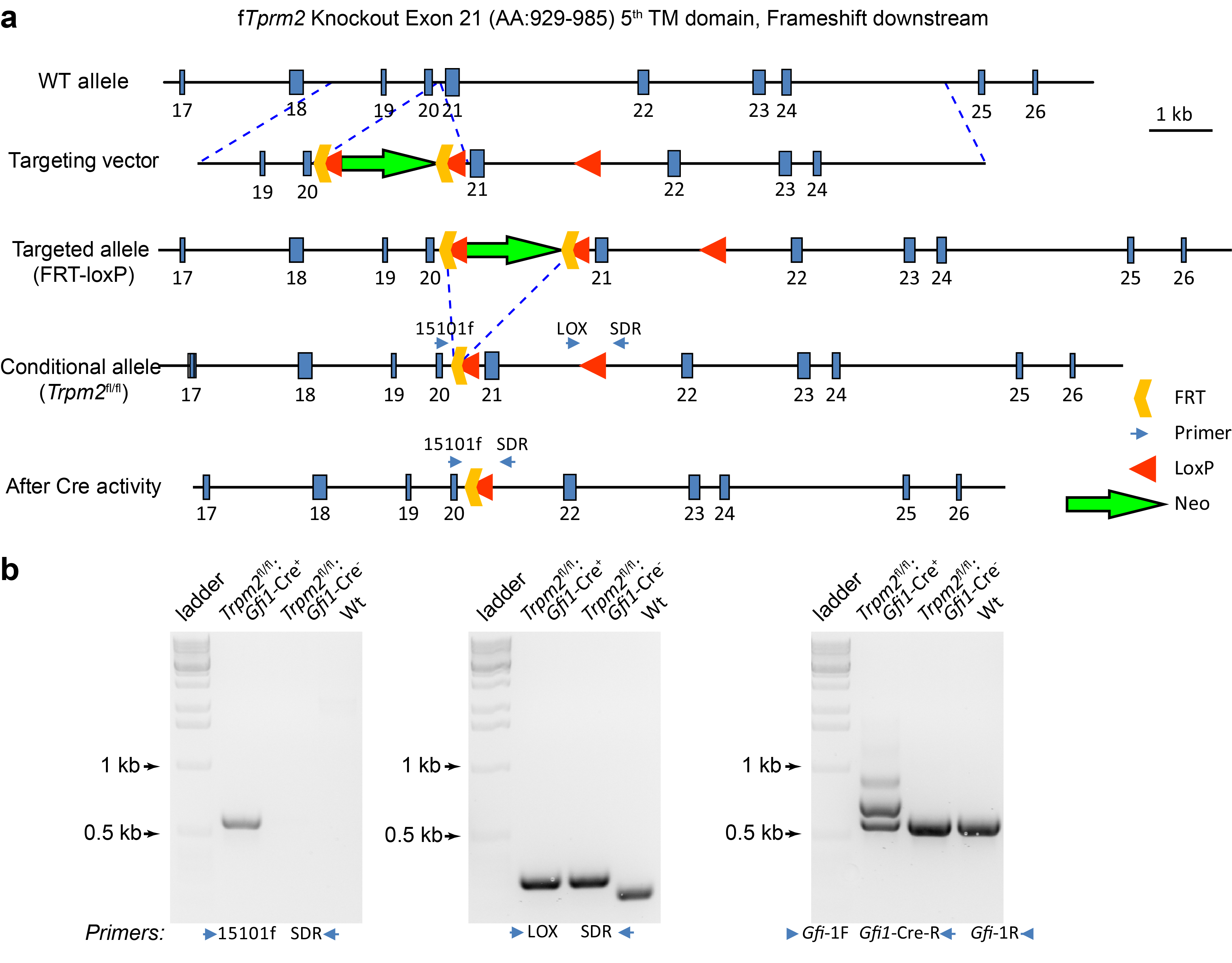

Supplement: S2 Fig — (a) Trpm2 conditional knockout strategy. We inserted two LoxP sites to flank a ~1.9 kb region that includes exon 21. A LoxP-FRT-Neo-LoxP-FRT cassette was inserted upstream of exon 21 and the third LoxP site was inserted downstream of exon 21. Exon 21 encodes aa929-985 including the essential fifth transmembrane domain and pore. The deletion also led to a downstream frameshift. Red arrowheads refer to LoxP sites, yellow brackets refer to FRT sites, and green arrows refer to the neomycin resistance gene. Primers used for genotyping and validation are indicated as gray arrows. (b) PCR from genomic DNA purified from inner ears of Trpm2fl/fl: Gfi1-Cre+ mice, Trpm2fl/fl:Gfi1-Cre- mice, and an age-matched wildtype mouse. (Left) PCR results using primer pair TM2cKO15101f and SDL. Lane 1 (Trpm2fl/fl:Gfi1-Cre+) shows a fused short band (616bp) only from the deleted allele; lane 2 lacking Cre (Trpm2fl/fl:Gfi1-Cre-) and Lane 3 (wildtype) do not show it. The fused band was confirmed by Sanger sequencing. (Middle) PCR bands at 320 bp (floxed allele) in lane 1 and lane 2, and 259 bp (Wt allele)in lane 3, using primers LOX and SDL, (Right) Genotyping for the Gfi1-Cre allele. A 1-kb DNA extension ladder was used (5 μl; Invitrogen #10511–012). (TIF) [file pone.0155577.s002.tif]

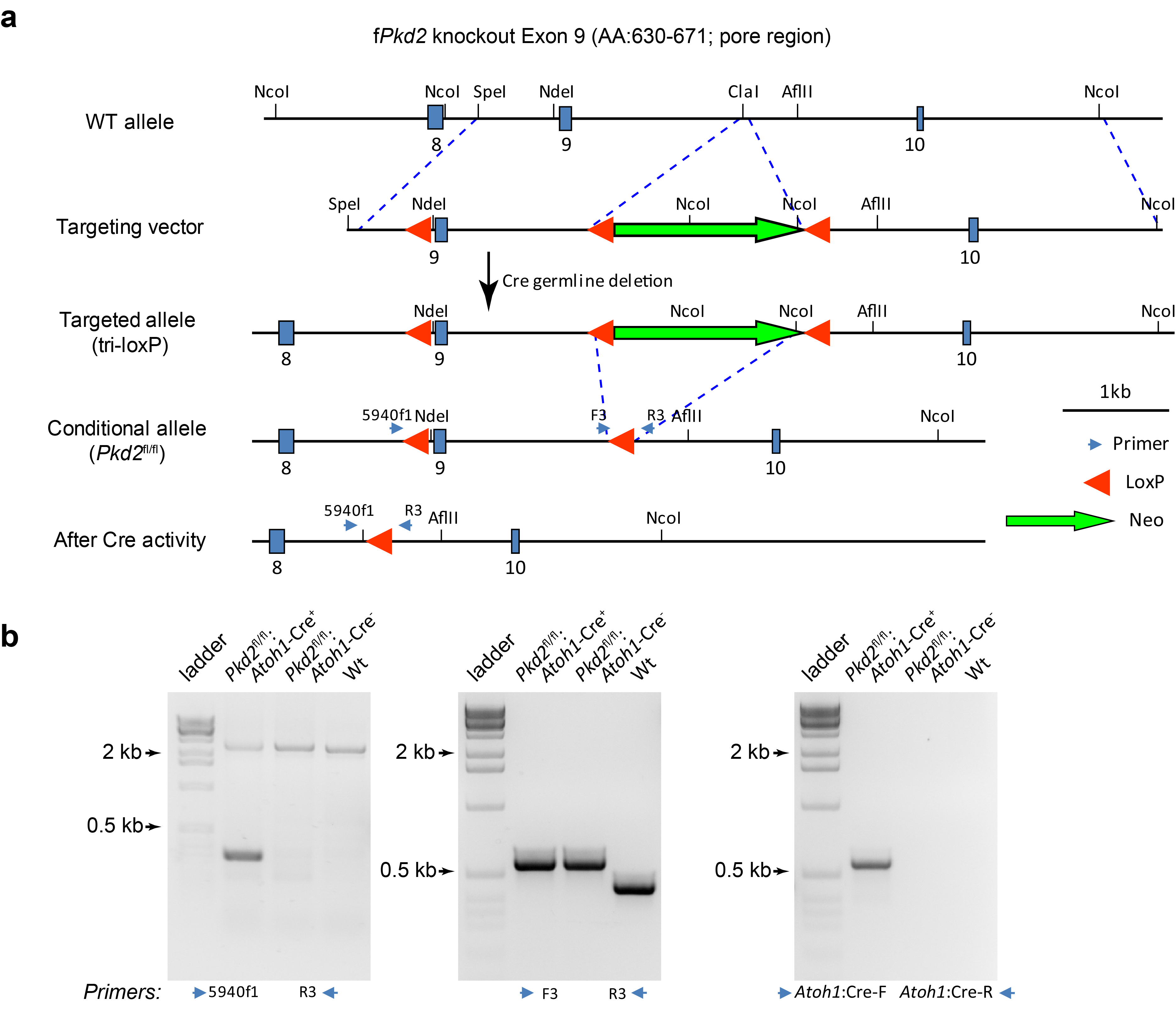

Supplement: S3 Fig — (a) Exon 9 of the Pkd2 gene was deleted by flanking the ~2.2 kb targeting region with two LoxP sites. A LoxP-Neo-LoxP cassette was inserted downstream of exon 9 and the third LoxP site was inserted upstream. Exon 9 encodes aa630-671 including the pore domain and most of the sixth transmembrane domain of the Pkd2 channel. Primers used for genotyping and validation are indicated as a gray arrows. (b) PCR from genomic DNA purified from inner ears of Pkd2fl/fl:Atoh1-Cre+ mice, Pkd2 fl/fl:Atoh1-Cre-/- mice, and an age-matched wildtype mouse. (Left) PCR results using primer pair PKD2-5940f1 and mPKD2in9R3. Lane 1 (Pkd2fl/fl:Atoh1-Cre+) shows a fused short band (344 bp) from the deleted allele and a longer band (>2437 bp) from the genomic DNA cells without Cre activity. The short fused band was confirmed by Sanger sequencing. Lane 2 (Pkd2 fl/fl:Atoh1-Cre-) shows the same long band as in lane 1 but no short band. Lane 3 shows a band of 2457 bp produced from a wildtype mouse inner ear. (Middle) PCR produced bands at 520bp in lane 1 and lane 2, and 468 bp in lane 3 using mPKD2in9F3 and mPKD2inR3. (Right) Genotyping of the Atoh1-Cre allele. A 1-kb DNA extension ladder was used (5 μl; Invitrogen #10511–012). (TIF) [file pone.0155577.s003.tif]

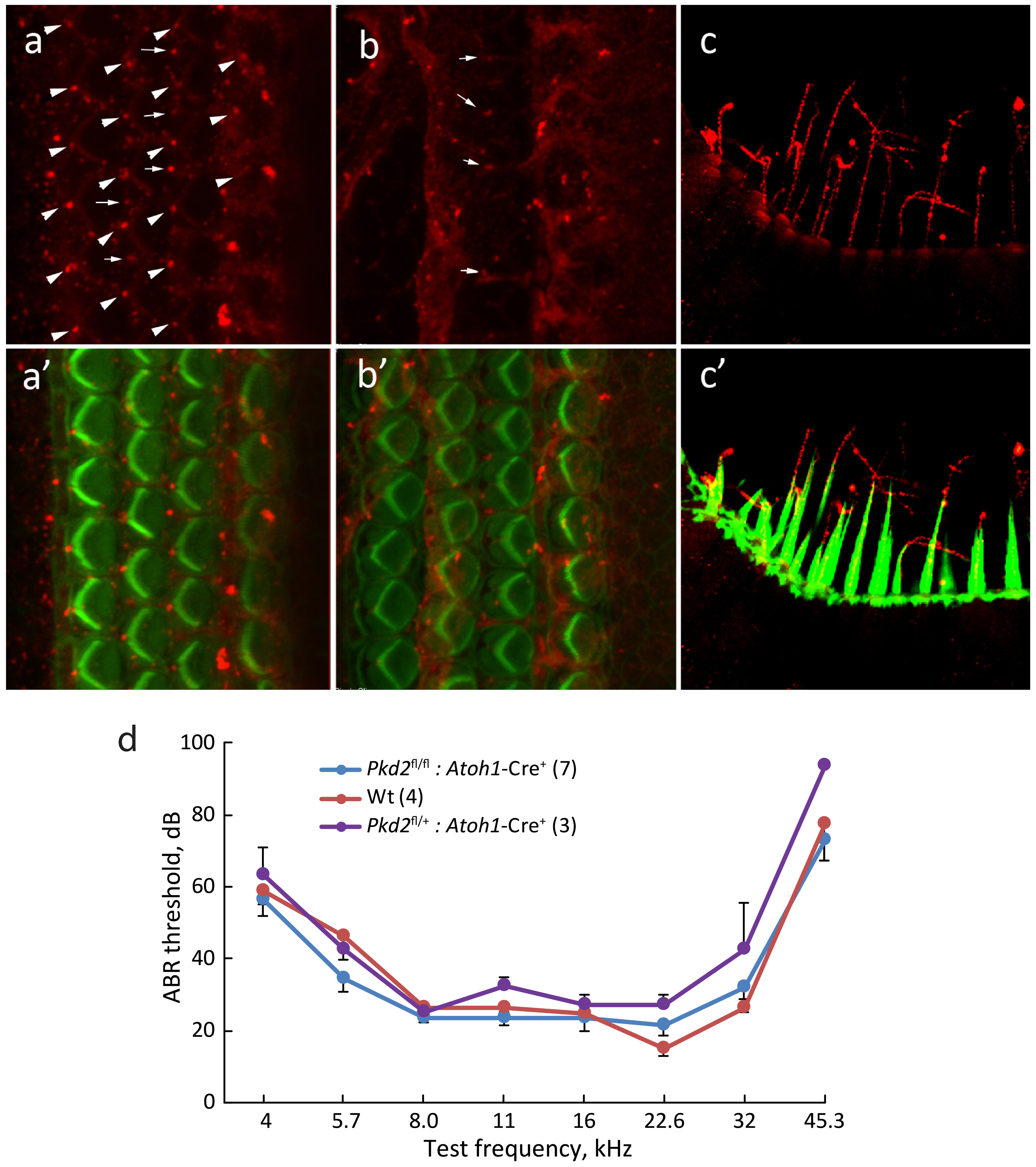

Supplement: S4 Fig — (a,a’) Antibody labeling (Santa Cruz Biotechnology, #sc-10376) for Pkd2 (red) and phalloidin staining for actin (green) in the heterozygote (Pkd2fl/+: Atoh1-Cre+) positive-control cochlea. Pkd2 label is evident in hair-cell kinocilia (arrowheads) and some supporting cell primary cilia (arrows). (b,b’) Pkd2fl/fl: Atoh1-Cre+ knockout cochlea. Pkd2 label is absent from hair cell kinocilia but not from supporting cell cilia. (c-c’) In vestibular hair cells of heterozygote mice, Pkd2 label is also in kinocilia. (d) ABR thresholds in response to pure tone stimuli. Pkd2fl/fl: Atoh1-Cre+ knockout mice show normal hearing. Data are mean ± SEM. (TIF) [file pone.0155577.s004.tif]

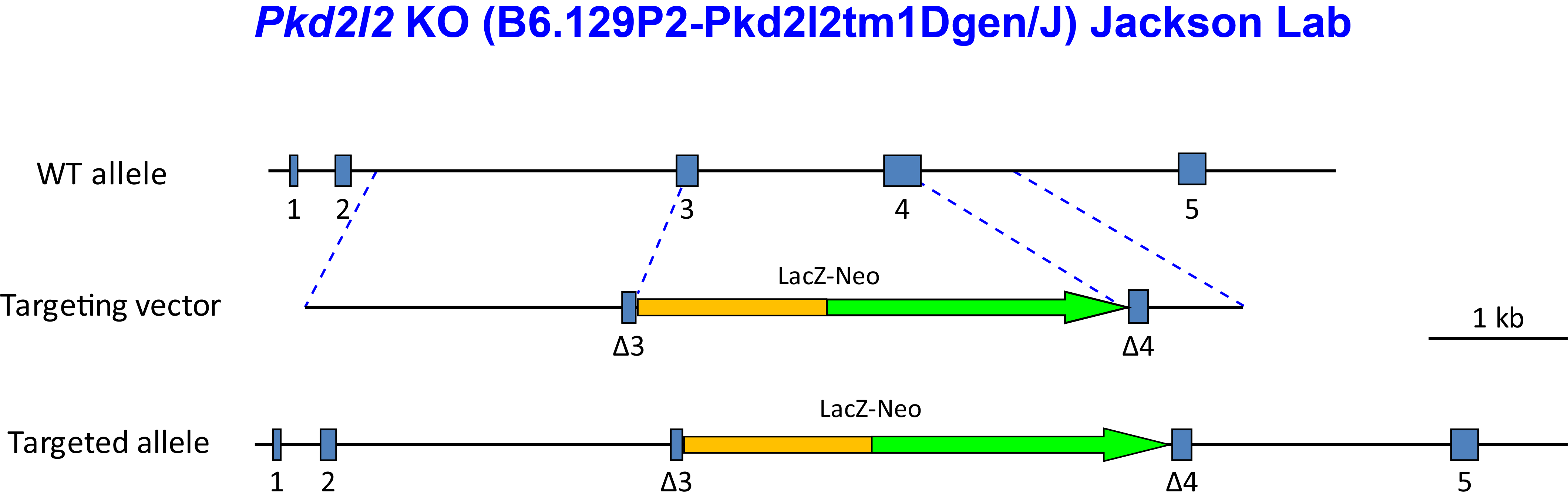

Supplement: S5 Fig — The generation of the Pkd2l2 knockout mouse, based on information from The Jackson Laboratory (Pkd2l2tm1Dgen/J; stock #005829; https://www.jax.org/strain/005829). A bacterial lacZ gene fused with a neomycin resistance gene replaced ~ 1.8 kb genomic sequence extending from the 3’ part of exon 3 to the 5’ part of exon 4. Thus the endogenous promoter drove the expression of beta-galactosidase. (TIF) [file pone.0155577.s005.tif]
